# MUT-7 Provides Molecular Insight into the Werner Syndrome Exonuclease

**DOI:** 10.3390/cells10123457

**Published:** 2021-12-08

**Authors:** Tsung-Yuan Hsu, Ling-Nung Hsu, Shih-Yu Chen, Bi-Tzen Juang

**Affiliations:** 1 Department of Biological Science and Technology, National Yang Ming Chiao Tung University, Hsinchu 300, Taiwan; tsungyuanhsu@gmail.com; 2 Department of Cell and Tissue Biology, University of California, 513 Parnassus, San Francisco, CA 94143, USA; 3 Occupational Safety and Health Office, Fu Jen Catholic University Hospital, New Taipei City 243, Taiwan; B01177@mail.fjuh.fju.edu.tw; 4 Institute of Biomedical Sciences, Academia Sinica, Taipei 115, Taiwan; sychen@ibms.sinica.edu.tw; 5 Center for Intelligent Drug Systems and Smart Bio-Devices (IDS2B), National Chiao Tung University, Hsinchu 300, Taiwan

**Keywords:** MUT-7, 3′-5′ exonucleases, *C. elegans* WRN-1 (CeWRN-1), small interfering RNA, Werner syndrome, *C. elegans* neuronal plasticity

## Abstract

Werner syndrome (WS) is a rare recessive genetic disease characterized by premature aging. Individuals with this disorder develop normally during childhood, but their physiological conditions exacerbate the aging process in late adolescence. WS is caused by mutation of the human WS gene (*WRN*), which encodes two main domains, a 3′-5′ exonuclease and a 3′-5′ helicase. *Caenorhabditis elegans* expresses human WRN orthologs as two different proteins: MUT-7, which has a 3′-5′ exonuclease domain, and *C. elegans* WRN-1 (CeWRN-1), which has only helicase domains. These unique proteins dynamically regulate olfactory memory in *C. elegans*, providing insight into the molecular roles of WRN domains in humans. In this review, we specifically focus on characterizing the function of MUT-7 in small interfering RNA (siRNA) synthesis in the cytoplasm and the roles of siRNA in directing nuclear CeWRN-1 loading onto a heterochromatin complex to induce negative feedback regulation. Further studies on the different contributions of the 3′-5′ exonuclease and helicase domains in the molecular mechanism will provide clues to the accelerated aging processes in WS.

## 1. Introduction

The maintenance of an intact genome is a complex process that depends on the fidelity of DNA replication, DNA secondary structure, and proteins that bind to DNA. Loss of these critical components may lead to the accumulation of chromosome mutations and impaired chromosome segregation, which gradually leads to the loss of cell function with age [1]. Cells have developed different mechanisms to enable the repair of most of this DNA damage and to preserve genomic content passed onto their offspring. 3′-5′ DNA exonucleases are key enzymes involved in many aspects of DNA repair and telomere maintenance and thus contribute to the maintenance of genome stability. Failure of DNA repair may trigger a major pathogenic mechanism leading to potential genetic disorders. However, the complexity and progression of a disease cannot be explained by DNA sequence variants. The study of progeroid syndromes has provided insight into how epigenetic regulation in genes triggers disease onset at different times. For example, Hutchinson–Gilford progeria syndrome (HGPS) is a classic childhood-onset premature aging disorder affected by pathogenic variants in the *LMNA* gene, which encodes the nuclear structural proteins Lamin A and C [2,3]. Nuclear Lamin and its nuclear envelope partners are involved not only in nuclear organization, serving as regulators of diverse nuclear processes, but also in the aging process, such as the mTOR (mechanistic target of rapamycin) signaling pathway [4,5]. Moreover, a mutation in the *LMNA* gene results in the accumulation of a truncated form of the dysfunctional protein progerin, which is the cause of early-onset HGPS. In a mouse model, progerin interacts with a subset of endoplasmic reticulum-associated proteins to cause metabolic abnormalities through changes in calcium homeostasis [6].

**Figure 1:**
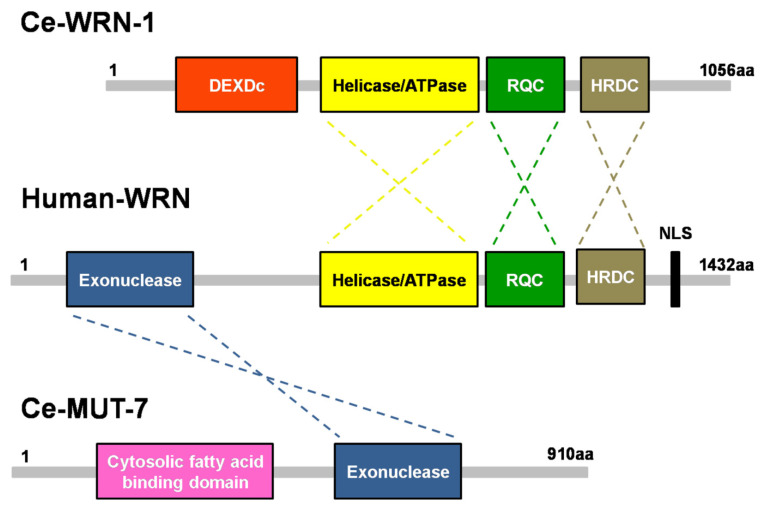
Domain structures of *C. elegans* MUT-7 and CeWRN-1 and human WRN. The 3′-5′ exonuclease domain of nematode MUT-7 has a 29% amino acid sequence identity to human WRN. The three helicase domains in the human WRN, helicase/ATPase, RecQ C-terminal domain (RQC), and helicase-and-RNaseD C-terminal (HRDC) domain, share 43%, 25%, and 16% identity, respectively, with the same domains of nematode WRN-1.

In a comparison of individuals starting to show aging, Werner syndrome (WS) is characterized by the adult-onset of the progeroid syndrome, and it is caused by mutations in the *WRN* gene, which encodes a multifunctional protein with 3′-5′ helicase and 3′-5′ exonuclease functions (Figure 1) [7]. Although data indicate that more than 400 mutations in *LMNA* have been identified as causative factors of diseases, including atypical WS, whose patients have tested negative for biallelic pathogenic WRN variants [8], classical WS patients show normal Lamin function. Previous studies with cell culture and mouse models have emphasized that the helicase domain of WRN proteins is not only important to DNA replication and DNA damage repair, but is also associated with several aging phenotypes. In HeLa cells, nucleolar localization of WRN requires HERC2, which possesses ubiquitin E3 ligase and acts in DNA replication and damage response [9]. Moreover, HERC2 inactivation inhibits rRNA transcription [9]. Loss of WRN helicase causes severe genome integrity defects in microsatellite high-instability cancer cells, and this functional impairment might be attributable to defective mismatch repair [10]. In molecular analysis of reprogrammed induced pluripotent stem cells (iPSCs) derived from WS fibroblasts, dysregulation of the PI3K/AKT pathway results in the impairment of angiogenesis, and this may be the reason for the poor healing of chronic ulcers and slow tissue regeneration observed in patients with WS [11]. Primary fibroblasts derived from WS patients show abnormal mitochondria including loss of cristae morphology, reduced density, and decreased cellular ATP levels compared to normal control fibroblasts [12]. Mitochondrial dysfunction is due to the inactivation of nicotinamide nucleotide adenylyltransferase 1 (NMNAT1), which is involved in NAD+ biosynthesis, suggesting a relationship between energy metabolism and aging. These phenotypes are also observed in model organisms, such as *C. elegans* and *Drosophila melanogaster* [12]. In a WS mouse model, mice lacking the helicase domain of the WRN ortholog display many WS features, including metabolic problems and a short life span. However, mice lacking the entire WRN protein do not show a premature aging phenotype [13]. This result implies different aging processes between humans and mice in WS.

The reasons that mutations in the WRN protein do not cause premature aging during childhood remain unclear. Recent studies on the function and structure of the WRN protein suggest the possibility of both 3′-5′ helicase and 3′-5′ exonuclease contributions to the premature aging process [12,14,15]. Misregulation of gene expression and epigenetic modification induced by mutations in the *WRN* gene are considered important factors contributing to the senescence process. Mesenchymal stem cells derived from WS patients show that the WRN protein associates with a heterochromatin complex including SUV39H1 and HP1 [16]. Moreover, a methylation array study between healthy individuals and WS patients showed 659 differentially methylated regions [17]. Here, we summarize some recently obtained comprehensive data showing the ways in which the helicase and exonuclease domains of WRN proteins contribute to different epigenetic regulatory mechanisms in a *C. elegans* animal model. These data may provide hints at the mechanisms through which siRNA synthesis and heterochromatin modification processes serve as potential determinants of adult-onset WS.

## 2. The Diverse Roles of 3′-5′ Exonucleases

The function of 3′-5′ exonucleases is hydrolyzation of phosphodiester bonds in nucleic acids; these exonucleases degrade the nucleic acid strand starting from the 3′ end. Although they were initially considered nonspecific and degradative enzymes, it has become clear that these exonucleases have distinct substrate affinities and play essential roles in a wide range of cellular processes. The 3′-5′ exonucleases are classified on the basis of their DNase and RNase activities, which depend on DNA or RNA targeted for binding and degradation. DNA exonucleases are required for the fidelity of DNA replication [18], DNA repair [19], telomere maintenance, and the stability of the nuclear genome [20]. In contrast, RNA exonucleases play multiple roles in regulating gene expression during the course of development or adaptation to environmental changes [21]. Since earlier studies have shown that the WRN 3′-5′ exonuclease can digest RNA and DNA strands [22], recent studies on this enzyme with DNase and RNase activity will be respectively described in the next section.

### 2.1. The Roles of 3′-5′ DNA Exonucleases

In normal cells, DNA replication in the S phase of the cell cycle is tightly regulated, leading to newly synthesized DNA strands with a low mutation rate and promoting cell viability and health. The accuracy of replication depends on three key processes: efficient selection of correct nucleotides in the DNA polymerization reaction, elimination of mistakenly incorporated nucleotides, and post-replication DNA mismatch repair [18]. The activation of DNA replication requires specific DNA helicases to open the double-stranded DNA helix. This opening allows DNA polymerases to duplicate the parental DNA strands in the 5′ to 3′ direction using RNA as a primer. When nucleotide misincorporation generates DNA base-base mismatches during DNA synthesis, the mismatched base is removed upon activation of the DNA mismatch repair process. Mismatch correction is a pathway highly conserved from *E. coli* to eukaryotic cells [23]. In general, a strand-specific nick is the starting point for the excision of a mismatched base. A specific helicase unwinds the DNA duplex, and DNA exonucleases move in either a 5′-3′ or a 3′-5′ orientation from the nick toward the mismatched base to the position adjacent to the mismatched base on the single-stranded DNA. The resulting single-strand gap is filled with the correct bases and sealed by DNA ligase.

During replication of the leading strand in eukaryotic cells, DNA synthesis continues to the end of the linear chromosome. In the lagging strand, DNA is synthesized in short stretches from different RNA primers. There is a problem with replication at the end of this strand: there is no space for an RNA primer; therefore, a new DNA fragment that includes the terminal nucleotides cannot be produced. As a result, the telomeres at the ends of chromosomes, which do not form Watson-Crick paired structures, are progressively shortened during each round of DNA replication. A special reverse transcriptase, a telomerase, resolves this problem by adding telomeric repeats to the ends of chromosomes. In human cells, TOE1, a 3′-5′ exonuclease, was recently shown to interact with the telomerase complex to regulate telomere maintenance [24]. Moreover, several human diseases, including WS, are associated with the dysfunctional replication of telomeres. There is evidence that WRN functionally and physically interacts with telomere-associated proteins TRF-2 and POT-1 to promote telomere instability [25,26,27]. Moreover, the WRN-TRF2 interaction mediates the 3′-5′ exonuclease activity of the WRN protein on DNA sequences containing telomeric repeats [28]. However, the mechanism by which WRN is involved in telomeric recombination events remains to be determined.

### 2.2. The Roles of 3′-5′ RNA Exonucleases

3′-5′ RNA exonucleases are essential for RNA metabolism in all cells, including the synthesis and degradation of coding and non-coding RNAs. First, 3′-5′ RNA exonucleases are involved in RNA processing that is critical for RNA maturation and stability. In eukaryotic cells, 3′-5′ RNA exonucleases trim the 3′ end of RNAs in the nucleus to yield mature noncoding RNAs [29,30,31]. These exonucleases are involved in the maturation of 5.8S ribosomal RNA (rRNA), small nuclear RNAs (snRNAs; such as U4 and U5), and small nucleolar RNAs (snoRNAs; such as U14, U18, and U24) [32,33,34]. In prokaryotic cells, 3′-5′ RNA exonucleases are involved in the 3′ end processing of rRNA and transfer RNA (tRNA). In *Pseudomonas syringae*, the 3′-5′ RNA exonuclease RNase R is required for 3′ end trimming of 16S and 5S rRNAs [35]. In *E. coli*, 3′-5′ RNA exonucleases, such as RNase T and RNase PH, contribute to the 3′ end maturation of tRNA [36,37]. Second, exonucleases, which degrade RNA, influence RNA expression levels, which are determined by the rates of RNA synthesis and degradation. Most mRNA degradation is associated with deadenylation performed by poly(A)-specific 3′-5′ RNA exonucleases [38]. Finally, 3′-5′ RNA exonucleases regulate the production of small RNAs such as microRNAs (miRNAs) and small interfering RNAs (siRNAs). The evolutionarily conserved 3′-5′ RNA exonuclease ERI-1 associates with the Dicer complex to generate certain siRNAs in *C. elegans* [39], and Eri-1 in mice has been shown to regulate global miRNA abundance [40]. Moreover, in *C. elegans*, forward genetic screens have revealed that the 3′-5′ RNA exonuclease activity of MUT-7 acts through small RNA-mediated silencing pathways [41].

## 3. Roles of the WRN Exonuclease in Gene Regulation

### 3.1. Functional Domains of the WRN Protein and Effects of Their Mutation

WS is a rare autosomal recessive inherited disease. Individuals with WS develop normally during childhood, but aging accelerates in late adolescence. The aging signs and symptoms include early graying and loss of hair, osteoporosis, atherosclerosis, cataracts, and type II diabetes mellitus [42,43,44]. Epidemiological studies have indicated that WS affects nearly 1 in 100,000 people worldwide. A higher frequency has been reported in Japan, where the incidence is 1 in 20,000 to 40,000 live births [45].

Most cases of WS have been linked to one or more mutations in *WRN*, which is located on chromosome 8p12 in the human genome [7]. The *WRN* gene encodes a 1432-amino acid protein with four functional domains (Figure 1, middle). WRN has a 3′-5′ exonuclease domain in its N-terminal region. The crystal structure of this exonuclease domain reveals that Mg^2+^-binding sites help to modulate exonuclease activity [15]. A helicase/ATPase domain and RecQ C-terminal domain (RQC) are located in the middle region of the protein. Biochemical analysis of the helicase has shown that ATPase activity produces the energy needed for unwinding double-stranded DNA with 3′-5′ polarity, suggesting that this enzyme may be involved in DNA replication, recombination, and repair [46]. A helicase and RNaseD C-terminal (HRDC) domain is found at the C-terminus. The HRDC domain has a weak DNA binding affinity but interacts with many different proteins [47]. In addition, a C-terminal nuclear localization signal (NLS) plays a critical role in importing WRN into the cell nucleus where it carries out its biological functions.

Based on functional domain characterization, WRN is considered a member of the RecQ helicase family [7,48]. The human genome contains five genes that encode RecQ helicases: *RecQ1*, *BLM*, *WRN*, *RecQ4*, and *RecQ5*. Mutations in *BLM* and *RecQ4* lead to Bloom syndrome and Rothmund–Thomson syndrome, respectively [48]. The *WRN* gene in WS patients often has nonsense or frameshift mutations that generate a stop codon, which leads to the production of a truncated WRN protein [49]. Furthermore, loss of the C-terminal NLS sequence means that truncated proteins fail to localize to the nucleus and are generally degraded in the cytoplasm [50,51,52,53]. Failure of WRN protein entry into the nucleus is considered to be the main pathogenic cause of WS because it results in changes in cellular homeostasis including DNA replication and stability.

### 3.2. Functions of the WRN Exonuclease

A large body of evidence has established a tight link between the human WRN protein and the regulation of DNA replication and repair. However, it remains unclear whether WRN has RNase activity or influences post-transcriptional modification. A recent study of human embryonic stem cells showed that WRN associates with a complex consisting of a trimethylated histone H3K9 (H3K9me3) methyltransferase, SUV39H1; a histone H3K9me3 binding protein, HP1α; and a nuclear envelope component, LAP2β [16]. This result may implicate epigenetic alterations in WRN-mediated premature aging. Interestingly, the function of the WRN exonuclease in epigenetic changes in humans is consistent with the role of the *C. elegans* WRN ortholog MUT-7 in olfactory learning processes. Olfactory learning requires downregulation of the expression of a guanylyl cyclase, ODR-1, by endogenous *odr-1* siRNA. The production of this siRNA is modulated by MUT-7 in the cytoplasm, and this siRNA is required for the formation of the complex with chromatin-associated heterochromatin protein 1 (HP1) homolog HPL-2 in the nucleus; HPL-2 binds to *odr-1* mRNA to silence ODR-1 expression in a negative feedback loop [54,55]. These findings suggest that the WRN exonuclease is critical for the installation of epigenetic modifications that regulate gene expression. The importance of WRN-like 3′-5′ exonuclease activity has been observed in other species. In *Arabidopsis thaliana*, loss of the WS-like exonuclease WEX causes defective posttranscriptional gene silencing [56]. In *Drosophila melanogaster*, loss of the fly WRN, which contains only a 3′-5′ exonuclease domain, affects lifespan via NAD^+^ supplementation [12].

In humans, 10–15% of patients with WS are nonclassical cases that present with the clinical manifestations of WS but possess the wild-type WRN gene [57]. Their pathogenic variant is found in mutations in the 3′-5′ exonuclease domain of POLD, which is a highly conserved human polymerase delta associated with the WRN helicase in lagging strand synthesis. In addition, some clinical case reports have described subjects who have mutations that cause a 90% reduction in WRN helicase activity but that do not affect exonuclease activity; these subjects do not develop clinical WS symptoms [14]. Thus, it is important to determine the roles of the helicase and 3′-5′ exonuclease domains of WRN to elucidate and distinguish the roles they play in WS.

## 4. Separate Functional Domains of the WRN Ortholog in *C. elegans*

Due to the complexity of human disease, a simple model organism that enables powerful genetic screening, such as *C. elegans*, can be helpful in identifying the mechanisms of a disease process [58,59]. *C. elegans* is a small soil-residing nematode with a short life cycle of 3.5 days and a lifespan of approximately 3 weeks at 20 °C. *C. elegans* homologs have been identified for 60–80% of human genes [60,61]. A search of the literature and the WormBase database (https://wormbase.org/, accessed on 13 October 2021) revealed that two nematode proteins are homologous to functional domains in the human WRN protein: CeWRN-1, which has an ATPase/helicase domain, and MUT-7, which has a 3′-5′ exonuclease domain (Figure 1, up and down panels). Loss of the nematode CeWRN-1 helicase leads to several progeroid signs, including decreased lifespan, cavity formation, and pharyngeal clogging in the worm head [62].

The exonuclease ortholog in nematodes was identified by RNA interference (RNAi) screening and transposon activity [41]. MUT-7 has been shown to participate in RNA regulation. Several reports have shown that MUT-7 is important for the amplification of secondary siRNAs that are produced after the initial siRNA stage [63,64,65]. Residues critical for MUT-7 3′-5′ exonuclease catalytic activity are conserved in the mammalian WRN exonuclease domain [41]. The WRN protein has both DNase and RNase activity [22], but few studies have focused on WRN RNA regulation, except to note that loss of the miRNA miR-124 in WRN-mutant mice seems to affect aging [66]. 

### 4.1. Olfactory Learning in C. elegans as a Model for WS

A hallmark of aging is the alteration of intercellular communication. In neurons, this communication is important for transferring correct information from sensory neurons, via interneurons, to motor neurons. Sensory neurons are at the frontline of neural networks and detect external stimuli. To respond correctly to a changing environment, the sensory system must be plastic enough to ignore prolonged stimuli that do not benefit the animal. This process is termed olfactory learning. The progressive loss of neuroplasticity is a sign of physiological aging.

**Figure 2:**
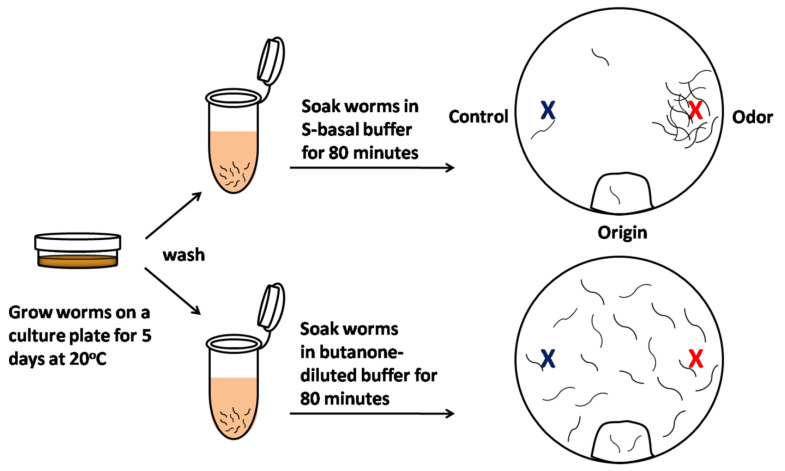
Scheme of olfactory learning assay in *C. elegans*. In the olfactory learning assay, olfactory behavior is quantified by the measurement of the chemotaxis index (CI). Five-day-old animals were cleaned to remove bacteria, half were pre-exposed to S-basal buffer alone (**top**), and half were pre-exposed to S-basal-diluted odor buffer, containing an odorant such as butanone (**bottom**). After the worms were incubated for 80 min, the animals were placed at the ‘origin’ of an assay plate containing an ethanol-diluted butanone spot (red) and a control ethanol spot (blue). The worms were allowed to roam around the dish for 120 min at 20 °C. The CI is the number of worms near the attractive odor subtracted from the number of worms near the control ethanol spot, divided by the total worms on the assay plate. These same worms can subsequently be screened to determine whether long-term olfactory learning occurs.

*C. elegans* is a good platform with which to model biological processes such as olfactory behavior because it relies extensively upon a sense of smell to not only detect food sources such as bacteria, but also to recognize odors that are not associated with food. *C. elegans* has 12 classes of amphid neurons with cilia exposed to the outside environment to enable the detection of environmental cues [67]. A pair of these neurons called amphid wing C (AWC) neurons detects attractive odorants, and their activity can be measured using a well-established chemotaxis assay (Figure 2). When the animal’s sensory neurons are stimulated by an odor, the neural response is reflected in its behavior. The animal’s naïve or primary response is to seek out innately attractive odors (chemotaxis; Figure 2 upper). When the animal is exposed to the previously attractive odor for a long time, this odor-seeking response is decreased, causing the animal to ignore the previously attractive odor (olfactory learning; Figure 2 lower).

**Figure 3:**
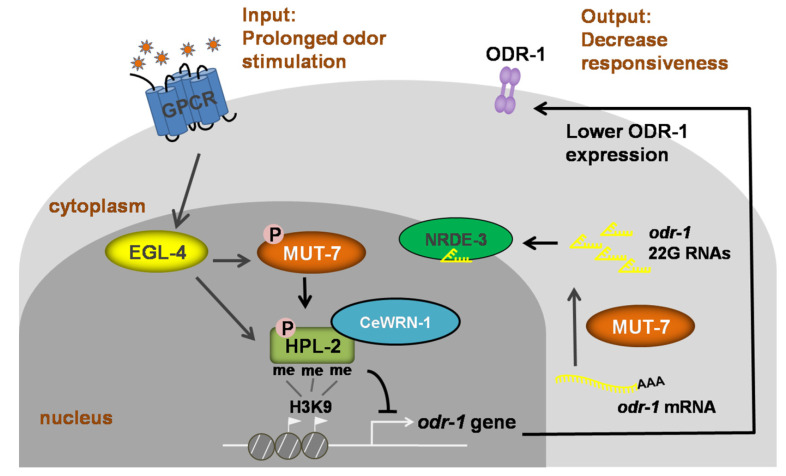
Signaling model of long-term olfactory learning in the AWC neuron of *C. elegans*. Wild-type AWC neurons enable the worm to detect an attractive odor and move toward it. When the worm is exposed to an odor for a long time, EGL-4 accumulates in the nucleus. Cytoplasmic MUT-7 mediates the synthesis of 22G siRNAs that target *odr-1* and the siRNAs are carried by NRDE-3 into the nucleus. Once the siRNA enters the nucleus, EGL-4 phosphorylates nuclear MUT-7, resulting in the association between CeWRN-1 and HPL-2. HPL-2 then binds to methylated histone H3.3, thereby downregulating *odr-1* transcription. Lower levels of the ODR-1 protein promote olfactory learning and worms ignore the previously attractive odor.

Chemotaxis and long-term odor learning in *C. elegans* are excellent models for studying behavioral plasticity and long-term memory behaviors at the molecular and genetic levels. A key behavioral “switch” involves the entry of the cGMP-dependent protein kinase EGL-4 into the nucleus of AWC neurons [68]. Prolonged exposure to the AWC neuron-sensed odors results in a decreased response to an odor that lasts for hours. This behavioral state is termed long-term olfactory learning and requires the accumulation of EGL-4 in the nucleus of AWC neurons [68,69]. When EGL-4 accumulates in the nucleus, MUT-7 acts specifically in the cytoplasm to promote the synthesis of 22G siRNA [54,55]. Endogenous 22G siRNAs are 22 nucleotides long and have a guanosine at the 5′ end [63]. The nuclear argonaute protein NRDE-3 acts as a shuttle to carry these 22G siRNAs from the cytoplasm to the nucleus [70]. The entrance of the 22G siRNAs into the nucleus induces nuclear EGL-4 to phosphorylate nuclear MUT-7 [54]. Activated MUT-7 is required for the association between HPL-2 and CeWRN-1 and their loading on the siRNA-targeted locus *odr-1* [54]. This results in a reduction in the mRNA level of *odr-1*, which encodes the guanylyl cyclase ODR-1 that generates the second messenger cGMP; it is this reduction that is highly correlated with the adaptation of the odor-seeking response [54,55]. These findings support the idea that the 3′-5′ exonuclease of MUT-7 is required for siRNA synthesis and suggest that the import of siRNA into the AWC neuron nucleus is required for CeWRN-1-dependent olfactory learning (Figure 3). This may provide hints about how small RNAs, such as miR-124, are differentially expressed in mice lacking WRN compared to wild-type mice [66].

### 4.2. C. elegans Model of WS Hypogonadism

A symptom of WS is hypogonadism of the testes in men and ovaries in women, which causes a reduction in fertility [49]. In *C. elegans*, although the loss of CeWRN-1 function seems to block the checkpoint function of DNA replication in the germline [71], the same mutant that lacks the helicase domain does not cause abnormalities in total brood size or germline cell death. In contrast, the loss-of-function *mut-7* mutant worm shows a significantly reduced total brood size compared to the egg number of wild-type worms [54]. A potential cause of this decreased brood size is the loss of germ cell viability. In support of this hypothesis, the number of germline cell deaths was high in the *mut-7*-null mutant [54]. The findings in *C. elegans* may open a new path to study the pathogenic mechanism of WS hypogonadism.

## 5. Conclusions and Future Directions

WS is a premature aging disorder that starts after adolescence. Currently, there is no cure for this disease, and clinical treatment options for WS only ameliorate symptoms. A therapeutic solution will require further development of an effective model organism and the use of this research platform to analyze the pathogenic mechanisms that underlie the symptoms of WS. Recent reports have indicated that human WS may not be caused by the loss of genome integrity, but rather by changes in epigenetic modifications [16,17]. *C. elegans* is a simple model organism, but powerful genetic screening has been applied to study the gene functions and signaling pathways of human neurodegenerative diseases [59]. The roles of the 3′-5′ exonuclease and helicase domains in the human WRN protein were initially clarified by examining spatial and temporal changes in *C. elegans* olfactory behavior, that is, chemotaxis and odor learning behavior after prolonged exposure to an odor. A number of questions have not been answered: First, the mechanism by which the 3′-5′ exonuclease and helicase domains of WRN dynamically regulate histone modification to shape neuronal plasticity should be studied. Research into siRNA-mediated regulation of the WRN-associated pathway is an emerging field. A complete catalog of all siRNAs and histone modifications will provide insight into the molecular mechanisms that drive the development of cellular plasticity. Second, it will be interesting to look for potential suppressors in the absence of a functional WRN protein. The neuronal-to-behavioral platform offered by *C. elegans* allows genome-wide screening in a relatively rapid and unbiased manner. Third, it has been challenging to devise novel therapeutic strategies for WS. Early phases of drug discovery and compound screening for this currently incurable disease may be achieved using this nematode platform [72].

Given the widely known association between cancer and WS, WRN is a specific target to develop a therapy for microsatellite instability-high (MSI-H) cancer cells [73]. WRN inactivation is selectively associated with the MSI-H status of cancer cells rather than the microsatellite stable (MSS) status of colorectal and endometrial cancer cell lines [10]. A relatively surprising finding was that gene expression analysis between WS cells and HGPS cells revealed very similar profiles [74]. Thus, a progerin inhibitor (SLC-D011) can improve the premature aging phenotypes of WRN iPSCs derived from fibroblasts and cardiac muscle cells [74]. Moreover, these iPSC lines derived from a WS patient have shown the potential to correct mutations in the WRN gene by using the CRISPR/Cas9-mediated method [75].

Multiple epigenetic changes may provide hints at the timing of onset and progression of WS since mutations in the WRN gene are not able to simply elicit adult premature aging. Recent studies have shown that epigenetic mechanisms could play an active role in driving the aging process. The ability of epigenetic systems may be added in a unique genomic position to change gene expression. It may be suggested that these systems translate the effects of various internal and external stimuli into molecular marks to change the rate of aging. Therefore, these genomic biomarkers in epigenetic mechanisms appear to be the most promising and effective strategy for developing WS therapies.

## Data Availability

Data sharing is not applicable to this review paper.
